# 3D printed fluidic swab for COVID-19 testing with improved diagnostic yield and user comfort

**DOI:** 10.1186/s40580-023-00393-3

**Published:** 2023-09-16

**Authors:** Joochan Kim, Jaehyung Jeon, Hyowon Jang, Youngkwang Moon, Abdurhaman Teyib Abafogi, Danny van Noort, Jinkee Lee, Taejoon Kang, Sungsu Park

**Affiliations:** 1 School of Mechanical Engineering, Sungkyunkwan University (SKKU), Seobu-ro 2066, Jangan-gu, Suwon, 16419 Korea; 2 Bionanotechnology Research Center, Korea Research Institute of Bioscience and Biotechnology (KRIBB), Gwahak-ro 291, Yuseong-gu, Daejeon, 34141 Korea; 3 Division of Biophysics and Bioengineering, IFM, Linköping University, Linköping, 58183 Sweden; 4 Department of Biophysics, Institute of Quantum Biophysics (IQB), Sungkyunkwan University (SKKU), Suwon, 16419 Korea; 5 School of Pharmacy, Sungkyunkwan University (SKKU), Suwon, 16419 Korea

**Keywords:** COVID-19, SARS-CoV-2, Mid-turbinate, Fluidic swab, 3D printing

## Abstract

The current standard method of diagnosing coronavirus disease 2019 (COVID-19) involves uncomfortable and invasive nasopharyngeal (NP) sampling using cotton swabs (CS), which can be unsuitable for self-testing. Although mid-turbinate sampling is an alternative, it has a lower diagnostic yield than NP sampling. Nasal wash (NW) has a similar diagnostic yield to NP sampling, but is cumbersome to perform. In this study, we introduce a 3D printed fluidic swab (3DPFS) that enables easy NW sampling for COVID-19 testing with improved diagnostic yield. The 3DPFS comprises a swab head, microchannel, and socket that can be connected to a syringe containing 250 µL of NW solution. The 3DPFS efficiently collects nasal fluid from the surface of the nasal cavity, resulting in higher sensitivity than CS for the detection of severe acute respiratory syndrome coronavirus 2 (SARS-CoV-2). This was confirmed by both reverse transcription-quantitative polymerase chain reaction (RT-qPCR) and lateral flow assays (LFA) in virus-spiked nasal samples and clinical samples. Additionally, users reported greater comfort when using the 3DPFS compared to CS. These findings suggest that the 3DPFS can improve the performance of COVID-19 testing by facilitating efficient and less painful nasal sample collection.

**Supplementary Information:**

The online version contains supplementary material available at 10.1186/s40580-023-00393-3.

## Introduction

Testing for coronavirus disease 2019 (COVID-19) has relied predominantly on nasopharyngeal (NP) sampling with cotton swabs (CS). However, this method requires healthcare professionals with personal protective equipment, limiting testing to specific sites such as health centers. COVID-19 self-testing has also used samples collected by CS, but self-testing often relies on samples collected from the mid-turbinate (MT) region of the nose, which is not as deep as the nasopharynx and may not contain high enough viral loads. Therefore, COVID-19 self-testing is considered less reliable than professional testing. In addition, the use of CS may impede the release of nasal mucus into the viral transport medium (VTM), leading to inadequate viral material for detection [1, 2]. Furthermore, many people are uncomfortable with CS-based NP sampling, and many countries have faced unprecedented CS shortages [3]. These issues underscore the need for improvements in sampling methods, including swab selection and sample type, for COVID-19 diagnosis.

Nasal wash (NW) is a technique of collecting nasal mucus by injecting sterile saline into the nostrils using a long tube, and it is considered an alternative to NP sampling [4]. NW samples can provide a comparable diagnostic yield to NP swab samples for the detection of severe acute respiratory syndrome coronavirus 2 (SARS-CoV-2) [5, 6]. However, the use of NW for COVID-19 testing is challenging because it requires the insertion of large volumes of NW solution into each nostril, which can be difficult for inexperienced individuals. To address the challenges of COVID-19 testing with NW, alternative sample collection methods have been explored. For example, three-dimensional (3D) printed swabs have been developed to collect nasal fluid (NF) through surface protrusions such as villi or bumps [7–9]. With 3D printing, it is possible to create swab shapes that are difficult to manufacture using traditional methods such as injection molding and micromachining. In addition, the 3D printed swabs could address the global CS shortage. More innovative 3D printed swabs are expected to be developed to improve the yield of sampling for COVID-19 testing.

**Fig. 1:**
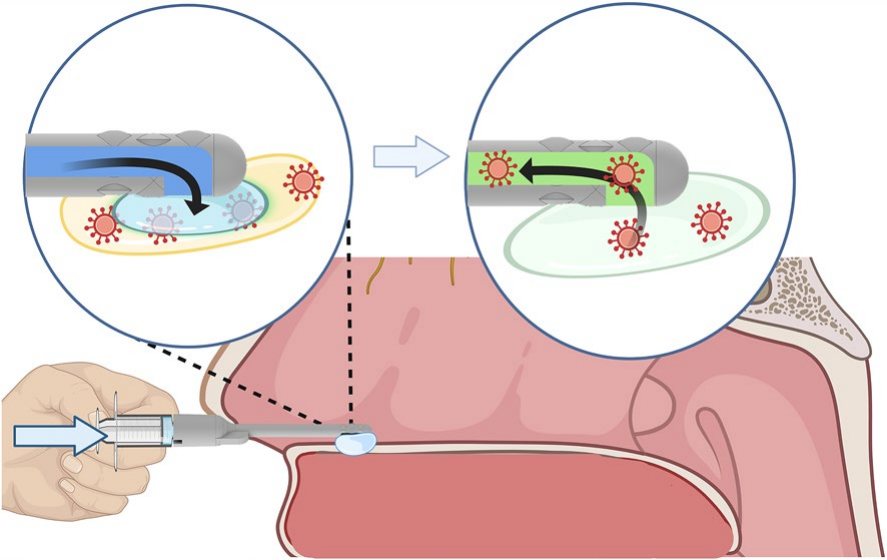
This schematic illustrates the process of self-collecting NF using the 3DPFS. The 3DPFS is inserted into the nasal cavity and the released NW solution flows into the MT, allowing for the subsequent collection of NF using the swab head with a single large hole. The 3DPFS consists of three parts: the swab head, microchannel, and socket, which are connected to a syringe filled with NW solution. Our study demonstrates that the 3DPFS can collect significantly more NF with relatively less pain compared to CS, making it a promising alternative for COVID-19 testing

In this study, we developed a 3D printed fluidic swab (3DPFS) to easily collect large amounts of NF from the MT region (Fig. 1) and verified that the detection sensitivity for SARS-CoV-2 is improved by using the 3DPFS. To maximize sampling yield, the 3DPFS was designed using computational fluid dynamics (CFD) simulation and fabricated to consist of a swab head with a single large hole, microchannel, and socket. The 3DPFS can be routinely connected to a syringe filled with NW solution, allowing it to efficiently rinse and collect NF from the surface of the nasal cavity. We demonstrated that the 3DPFS collected a significantly greater amount of NF than CS. The feasibility of 3DPFS for COVID-19 testing was validated by using SARS-CoV-2 lysate-spiked artificial NF and COVID-19 patient nasal samples (PNS). Both 3DPFS and CS were used to collect NF from the samples, followed by reverse transcription-quantitative polymerase chain reaction (RT-qPCR) and lateral flow assays (LFA). Notably, the 3DPFS showed higher sensitivity than CS, showing that 3DPFS is an improved NF sampling tool for SARS-CoV-2 detection. Moreover, a user survey showed that the 3DPFS caused less pain than CS during NF collection. The use of 3DPFS to improve sensitivity for SARS-CoV-2 detection may also have broader implications in the development of more efficient and accurate diagnostic tools for other infectious diseases in the future.

## Methods/experimental

### Materials

100% glycerol, trypsin, tris(2-carboxyethyl)phosphine hydrochloride (TCEP), and ethylenediaminetetraacetic acid (EDTA) were purchased from Sigma-Aldrich (St. Louis, MO, USA). Navy-blue dye was purchased from Chefmaster (Fullerton, CA, USA). Phosphate buffered saline (PBS, pH 7.4) was purchased from Gibco (Grand Island, NY, USA). CS were purchased from Soosung (Yangsan, Korea). NW solution (0.9% NaCl) was purchased from Medicore (Pocheon, Korea). NF Samples with normal viscosity were generated using artificial NF (Biochemazone; Edmonton, AB, Canada), without any additional substances. This artificial NF was selected due to its representation of normal viscosity. Conversely, NF samples with high viscosity were prepared using 100% glycerol, chosen for its viscosity similarity to that found in patients with symptomatic conditions [10, 11].

### Fabrication of 3DPFS

**Fig. 2:**
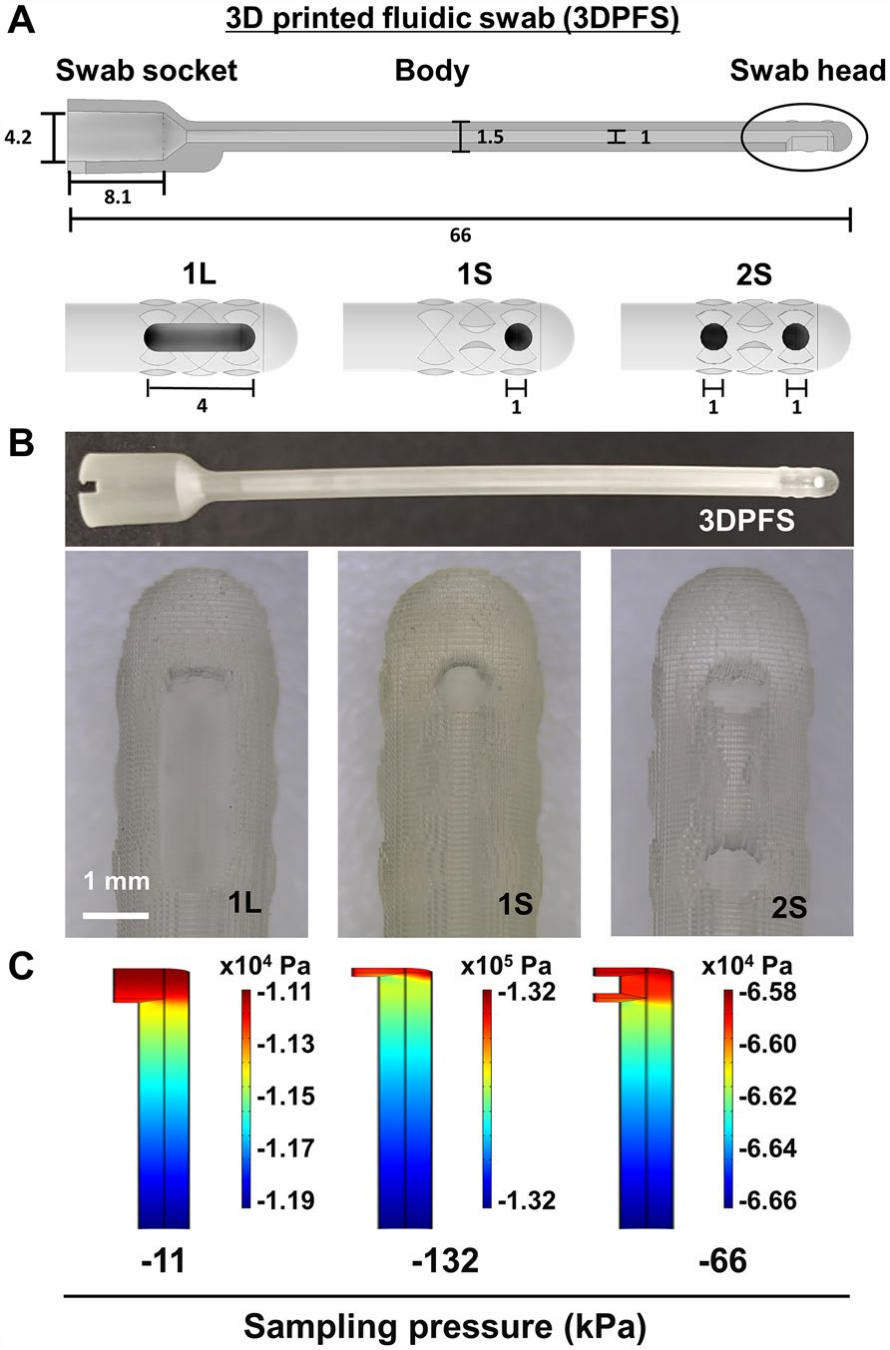
Dimensions and designs of the 3DPFS swab heads, as well as the results of CFD simulations that were conducted to evaluate the sampling pressure required for each swab head design. **A** Schematic representations of the three swab head designs, 1L, 1S, and 2S, with different hole geometries. 1L has a rounded rectangular hole (4 mm by 1 mm), while 1S and 2S have a single and two small round holes (1 mm in diameter), respectively. L denotes a large hole, and S denotes a small hole. **B** Actual images of the 3DPFS and its three swab head designs. **C** CFD simulation results indicating that the required sampling pressure for each model was different, with − 11.9 kPa for 1L, − 132.5 kPa for 1S, and − 66.6 kPa for 2S

The 3DPFS was designed using Autodesk Inventor 2021 Student version (San Rafael, CA, USA) and printed using a digital light processing (DLP) 3D printer IM1^™^ (Carima, Seoul, Korea) with photocurable resins CUKH010C (Carima) at 405 nm wavelength. It consists of swab head, body, and socket (Fig. 2A), and has a total length of 66 mm. The swab head features holes and protrusions for fluid exchange and collection. The channel in the body part has a diameter of 1 mm and a length of 50.5 mm, allowing fluid to flow through. The outer diameter of body is 1.5 mm, which minimizes discomfort during insertion into the nose. The socket, with an inner diameter of 4.2 mm and a length of 8.1 mm, is designed to fit to the head of a 1 mL syringe (DeayoungLab, Seoul, Korea).

The 3D printing parameters are provided in Additional file 1: Table S1. After printing, the 3DPFS was removed from the printing plate and immersed in a 70% ethanol solution for 10 min. The surface of the swab was thoroughly washed and attached to a 1 mL syringe. The channel inside was washed 3 times with the ethanol solution using the syringe and then washed 2 times with distilled water. The swab was then detached from the syringe, dried at 70 ℃ in an MCO-15AC oven (SANYO Electric Co., Osaka, Japan), and post-cured with ultraviolet (UV) light for 30 min, as recommended by the manufacturer.

### CFD simulation

We created 3D numerical models of the geometry using SolidWorks (Dassault Systems, Vélizy-Villacoublay, France). The pressure, the velocity of the fluid, and the motion of NF were calculated by Multiphysics version 5.4 (COMSOL Inc., Burlington, MA, USA). The NF is a non-Newtonian fluid that is incompressible, and we measured its viscosity using the HR30 rheometer (TA Instruments, New Castle, DE, USA). The shear-thinning Carreau model was used as follows:

$$\mu \left(\dot{\gamma }\right)={\mu }_{\infty }+\frac{{\mu }_{0}-{\mu }_{\infty }}{{\left(1+{\left(\lambda \dot{\gamma }\right)}^{2}\right)}^{a}}$$

where $\mu \left(\dot{\gamma }\right)$ is the viscosity of the NF, *μ*_*0*_ is the viscosity in the zero-shear rate condition, *μ*_*ꝏ*_ is the infinite limit viscosity, $\dot{\gamma }$ is the shear rate defined as $\nabla \mathbf{u}+{\left(\nabla \mathbf{u}\right)}^{\mathrm{T}}$, $\mathbf{u}$ is the flow of the NF, *λ* is a characteristic time, and *a* is constant related to the type of fluid; shear-thinning fluid $(a<0)$, Newtonian fluid $(a=0)$, and shear-thickening fluid $(a>0)$ [12]. The parameters *μ*_*ꝏ*_, *μ*_*0*_, and $a$ for the model were determined to be 5.14 × 10^–3^ Pa⋅s, 20.99 Pa⋅s, and − 0.5, respectively.

The Navier–Stokes equation was used for the calculation of the fluid velocity and pressure. The NF has a low Reynolds number from 4.68 × 10^–3^ at *μ*_*0*_ to 191.09 at *μ*_*ꝏ*_ in the flow channel so that the flow of the NF followed the continuity equation and the momentum equation of Navier–Stokes equation for laminar flow.

The continuity equation is given as:

$$\rho \nabla \bullet \mathbf{u}=0$$

The momentum equation is expressed as:

$$\rho \left(\frac{\partial \mathbf{u}}{\partial \mathrm{t}}+\left(\mathbf{u}\bullet \nabla \right)\mathbf{u}\right)=-\nabla p+\rho {\varvec{g}}+\mu {\nabla }^{2}\mathbf{u}$$

where *ρ* is the density of the NF (= 982.2 kg m^−3^), and ${\varvec{g}}$ is the gravitational acceleration. The water was loaded in the flow channel before collecting the NF and it also followed the same equation with the NF because the Reynolds number of the water was 997.

The NF is composed of 95 wt.% water, 5 wt.% mucin glycoprotein, and minor components such as electrolytes [13]. The NF was mixed with water by advection and diffusion when it was collected using the 3DPFS. The convection–diffusion equation is represented as:

$$\frac{\partial c}{\partial t}+\mathrm{u}\bullet \nabla c={D}_{AB}{\nabla }^{2}c$$

where *c* is the concentration of mucin, and ${D}_{AB}$ is the diffusivity which is 9.42 × 10^–12^ m^2^/s of mucin (solute, A) for water (solvent, B). The initial concentration of the NF is 5 wt.%. The NF diffusivity at room temperature (RT) was calculated by Polson’s equation used for a molecular weight above 1000 g mol^−1^ and Polson’s equation is expressed as [14]:

$${D}_{AB}=\frac{9.40\times {10}^{-15}T}{{\mu }_{B}{M}_{A}^{1/3}}$$

where *T* is the temperature, ${\mu }_{B}$ is the viscosity of water, and ${M}_{A}$ is the molecular weight of mucin. The values of *T*, ${\mu }_{B}$, and ${M}_{A}$ are 293.15 K, 0.001 Pa⋅s, and 25 × 10^6^ g/mol, respectively [15].

A grid independent test was performed to find the optimal number of meshes for accurate calculation of the simulation. Grid independence tests were performed on 4 different grid distributions for each 3DPFS model; one large hole: 26948, 54979, 178721 and 362827, one small hole: 22506, 48730, 157754 and 320197, and two small holes: 24871, 50888, 164011 and 333233. The simulation results did not change when using more than approximately 150000 grids.

### Sampling yields of NF in flat surface by 3DPFS and CS

To compare the sampling yield of the 3DPFS and CS, the amount of NF collected from slide glasses was calculated through dye absorption analysis. For this analysis, 15 mL of artificial NF was mixed with 150 µL of navy-blue dye, and 100 µL of the blue NF was gently dropped onto a slide glass and collected using either 3DPFS or CS.

Before collecting the NF from the surface of a slide glass, the 3DPFS was connected to a 1 mL syringe filled with 250 µL of NW solution through its socket. By gently pressing and withdrawing the plunger of the syringe attached to the 3DPFS, the NF on the surface was rinsed and collected with released NW. The collected NF along with 250 µL of NW in the 3DPFS was released into a microtube containing 150 µL of NW solution.

The blue NF on a slide glass was collected using a CS and the CS was dipped into a microtube containing 400 µL of NW solution for 15 min at RT and was vortexed for 5 s to fully release the NF into the NW solution.

To estimate the sampling yield from the 3DPFS and CS, 100 μL of collected NW solution in the microtubes was taken and mixed into a cuvette containing 900 μL of distilled water. The color intensity of the cuvette was measured at 600 nm using a cell density meter WPA CO8000 (Biochrom Ltd, Cambridge, England) to calculate collected volume (μL) by the 3DPFS or CS using the following equation [16]:

$$\mathrm{Collected\, volume }\left(\mathrm{\mu L}\right)=\frac{{m}_{f}-{m}_{i}}{\rho }$$

where, ${m}_{f}$ stands for mass of final swab after collection and ${m}_{i}$ stands for mass of the initial swab before collection. The density of the sample is represented by *ρ*.

Released volume (μL) was calculated as reported previously [17]:

$$\mathrm{Released\, volume }\left(\mathrm{\mu L}\right)=\frac{microtub{e}_{f}-mircotub{e}_{i}}{\rho }$$

The collected volume is released to microtube that contains 400 μL of NW solution. Therefore, initial weight of microtube ($mircotub{e}_{i}$) was deducted from the weight of the final microtube ($microtub{e}_{f}$) after release of collected sample to the tube to find the released amount. Absorbance at 600 nm (%) was calculated as:

$$\mathrm{Absorbance \,at }\,600\mathrm\,{ nm }\left(\mathrm{\%}\right)=\frac{Ab{s}_{swab}}{Ab{s}_{control}}\times 100\mathrm{\%}$$

$Ab{s}_{swab}$ was calculated reverse calculating the standard curve to find how much portion of the control was presented in the final volume.

### SARS-CoV-2 lysates

SARS-CoV-2 (BetaCoV/Korea/KCDC03/2020) was provided by the National Culture Collection for Pathogens (NCCP), which is overseen by the Korea National Institute of Health. The virus sample was processed at the Biosafety Level 3 (BL-3) facility of the Korea Research Institute of Bioscience and Biotechnology (KRIBB), which is approved by the Korea Centers for Disease Control and Prevention (KCDC). In detail, 90 μL of virus samples were combined with 10 μL of TCEP/EDTA (final concentrations of 100 and 1 mM, respectively). The mixture was then heated to 50 ℃ for 5 min and to 64 ℃ for 5 min.

### SARS-CoV-2-spiked NF

The viral lysates were diluted using PBS from 10^5^ to 10 plaque forming unit (pfu)/mL and 100 μL of each diluted lysate was mixed with 900 μL of artificial NF.

### COVID-19 PNS

A total of eight samples were obtained from Gyeongsang National University College of Medicine. In detail, NP aspirates were collected from patients using flocked NP swabs and were placed in virus transport media (UTM, Copan Diagnostics Inc., Murrieta, CA, USA). Samples were then stored at − 85 ℃ until use. The protocol was reviewed and approved by the Institutional Review Board of Gyeongsang National University College of Medicine, Jinju, Korea (IRB approval number: 2022-10-012) prior to the commencement of this study.

### Collected amounts of SARS-CoV-2-spiked NF on slide glasses by 3DPFS and CS

The viral lysates-spiked NF (100 μL) was gently dropped to a slide glass and the NF was collected using the 3DPFS or CS as described above in dye absorption analysis. For 3DPFS, 250 μL of NW was used to collect the NF and was released into a microtube containing 150 μL of NW solution, while the CS was dipped into a microtube containing 400 μL of NW solution after collecting the NF. The viral load of the collected NF was estimated using both RT-qPCR and LFA.

### Collection yields of SARS-CoV-2-spiked NF and PNS from 3D printed human nose by 3DPFS and CS

To compare the performance of the 3DPFS and CS for collecting NF from the human nose, we added 100 μL of virus-spiked NF into the nasal cavity of a 3D printed human nose model, located approximately 2 cm from the nostril. We used a disposable pipette to deliver the NF through one of the nostrils into the MT (Additional file 1: Fig. S1). The NF from the nostril was collected by the 3DPFS or CS and tested with LFA.

Similarly, 50 μL of PNS was added into the MT and was allowed 15 min of evaporation at RT before the collection. The viral ribonucleic acid (RNA) of the collected PNS was quantified using RT-qPCR.

The 3D CAD design for human head was found elsewhere [18]. The design was printed by a DLP 3D printer (TM200^™^, Carima) with the standard beige photopolymer resin (3DK83B) from Carima. The printing parameters for the 3D printing are listed in Additional file 1: Table S2.

### RT-qPCR

RNAs of SARS-CoV-2-spiked NF and PNS were extracted using High Pure Viral RNA Kit (Roche, Mannheim, Germany). RNA extractions were carried out in accordance with the manufacturer’s standard protocol. RT-qPCR was then performed using Takara One-Step TB Green^®^ PrimeScript^™^ RT-PCR Kit II (Kusatsu, Shiga, Japan) according to the manufacturer’s instruction. Briefly, extracted RNA was combined with RT-qPCR components and open reading frame (ORF) gene primers, which then underwent reverse transcription for 5 min at 42 ℃, PCR for 10 s at 95 ℃, and 50 cycles of 5 s at 95 ℃ and 30 s at 60 ℃. The CFX opus Real-Time System (Bio-Rad) was used to measure the fluorescent signal during each cycle of the PCR step, and the system’s built-in software (CFX maestro) was utilized to obtain the threshold cycle (C_t_) values. The following is the sequence of the primers that were used to detect the ORF gene. Forward primer: 5′-TTC TGC TGC TCT TCA ACC TGA-3′, Reverse primer: 5′-ATA GTC TGA ACA ACT GGT GTA AGT-3′ [19].

### LFA

COVID-19 Self-diagnostic kit (Ag Home Test) from GenBody (Cheonan, Korea) was used for the experiments. In brief, 250 µL of NW was used to collect NF by the 3DPFS and was then released to a tube with 150 µL of diluent provided by the manufacturer, while the CS after collection was dipped into 400 µL of the diluent and swirled to release the collected NF. Four drops of the diluent containing the collected NF were dropped into the sample loading zone of the LFA.

### Analysis of color intensity of LFA

The self-diagnostic kit has two lines, control and test lines. To quantify the amount of viral load in the sample, the color intensity of each line was measured using ImageJ (NIH, Bethesda, MD, USA). The background noise was removed by drawing a trendline at the bottom (Additional file 1: Fig. S2) and the color intensity of each line was calculated by measuring the area between the peak and the trendline. The test line intensity was normalized by dividing it by color intensity of the control line from the same device.

$$Normalized\, intensity \,of\, test\, line \left(\mathrm{\%}\right)=\frac{ intenstiy\, of \,the \,color\, of \,the\, test \,line}{intensity \,of\, the \,color \,of \,the \,control\, line }\times 100\mathrm{\%}$$

### User survey for easiness and comfortability of 3DPFS and CS

A survey was conducted among 28 participants who had prior experience with COVID-19 testing at a testing center using NP CS (IRB approval number: 2022-01-012-001). Participants were given instructions on how to use the 3DPFS and were asked to collect samples using the 3DPFS on their own. The survey was administered immediately after sample collection and aimed to compare the ease and comfort of using the 3DPFS versus CS. The questionnaire used in the survey to assess participants’ pain, discomfort, and preference between CS and the 3DPFS for COVID-19 testing can be found in Additional file 1: Fig. S3.

### Statistical methods

Most of the data shown were based on the mean ± standard deviation of three independently performed experiments. Student’s t-test was used to compare the data obtained under different conditions. Data with a *p*-value less than 0.05 were considered significant. *p < 0.05, **p < 0.01, ***p < 0.001. Participants, data or tissue or animals must include statement on ethics approval and consent.

## Results and discussion

### Design and optimization of 3DPFS

The 3DPFS (Fig. 2A) has a relatively short length of 6.6 cm compared to the NP swab, which typically requires an insertion depth of 9.40 ± 0.64 cm [20]. Its swab head features holes for fluid exchange, positioned on the side to keep it submerged in liquid during sample collection. This design ensures that the 3DPFS can effectively collect fluids without drawing in air, which would interfere with the collection process. A fin-like structure near the swab socket helps the user to orient the device, while protrusions on the swab head aid in collecting samples from various surfaces within the nasal cavity (Fig. 2A, B). Unlike other 3D printed swabs [7–9, 21, 22], which rely on their porous surfaces to collect NF, the 3DFPS was designed to take advantage of the property of NW solution that lowers the viscosity of NF.

The design of the swab head plays a critical role in the fluid dynamics of the 3DPFS, as it determines the area through which fluids can flow. To optimize the swab head design, three different models (1L, 1S, and 2S) were created, with varying hole geometries. 1L had a rounded rectangular hole (4 mm by 1 mm), while 1S and 2S had a single and two small round holes (1 mm in diameter), respectively. According to the results of the CFD simulation (Additional file 1: Fig. S4), the fluid velocity and volume fraction were found to be similar for all three swab head models. However, there were differences in the required sampling pressures, with 1L requiring the lowest pressure of -11.9 kPa, followed by 2S at − 66.6 kPa, and 1S requiring the highest pressure of − 132.5 kPa (Fig. 2C). These findings suggest that 1L may be more effective in collecting high-viscosity samples, including NF, due to its larger opening, which requires the lowest force for sample collection. We also attempted to use a fourth model, “6S,” which had six small circular holes arranged in three different directions (Additional file 1: Fig. S5). However, this model was unsuccessful as it relied solely on surface tension for fluid collection and did not have sufficient porous area to capture any solution. Based on the simulation results, we selected the 1L model as the optimized 3DPFS for collecting NF.

The design of the swab necessitated a slim profile to ensure comfortable insertion into the nasal cavity. Consequently, thicker designs were unsuitable for substitution. Moreover, the swab was characterized by a tubular structure, demanding robust walls. The 3D printer's minimum resolution dictated the fabrication of a swab with an outer diameter of 1.5 mm and a central hole diameter of 1 mm. These dimensions effectively balanced successful printing outcomes with the structural resilience essential for enduring stress. As a result, the tubes possess a hole width of 1 mm. Initial prototypes encompassed the 1S and 2S models. Subsequently, the 1L model was conceived, which enlarged the central hole by extending the space between the two orifices of the 2S version. While exploring elongated hole lengths holds potential for enhanced functionality, this aspect remained unexplored in the current study.

### Amount of NF from slide glasses by 3DPFS and CS

**Fig. 3:**
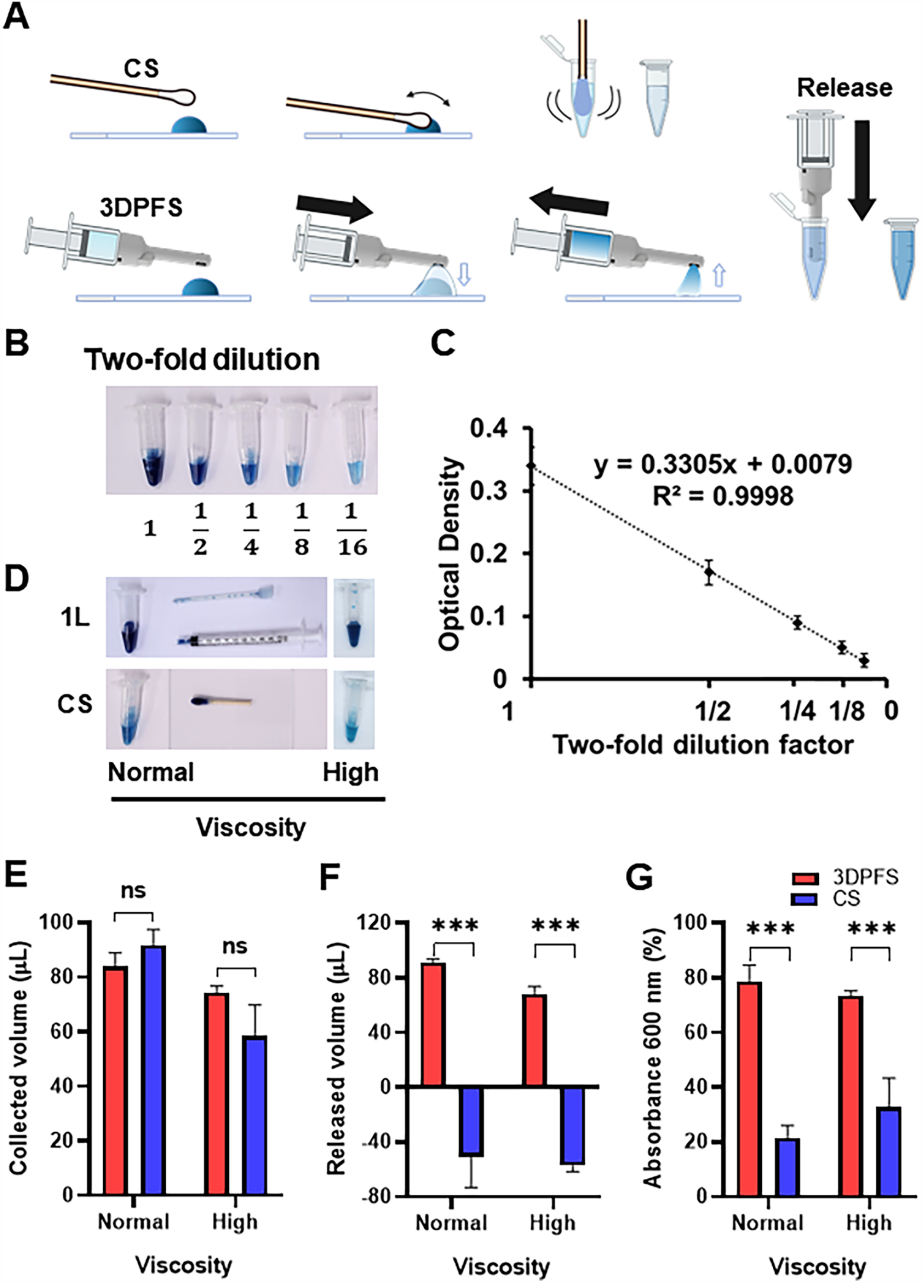
Collected and released volume of NF on a flat surface by the 3DPFS and CS. **A** Schematic diagram illustrating the process for collecting 100 µL of NF containing blue dye on slide glasses using the 3DPFS and CS. The collected NF was released into a microtube for analysis of collected and released volumes by dye absorption. **B** Serial two-fold dilution of NF containing blue dye. **C** Standard curve drawn from optical density of the diluted NF samples shown in (**B**). **D** Comparison of NF collection efficiency between the 3DPFS and CS at different viscosities. © Collected volume of NF samples with normal and high viscosity by the 3DPFS and CS. **F** Released volume of NF samples with normal and high viscosity by the 3DPFS and CS. **G** Absorbance at 600 nm of the NF samples collected and released by the 3DPFS and CS. The data were based on the mean ± standard deviation of three independently performed experiments. Statistical analysis was performed using Stud’nt’s t-test, and data with a *p*-value less than 0.05 were considered significant (*p < 0.05, **p < 0.01, ***p < 0.001). n = 3

To compare the ability of the 3DPFS and CS to collect and release NF, we used blue dye mixed with artificial NF. One hundred µL of this fluid was dropped onto a slide glass and collected it with both the 3DPFS and CS, as shown in Fig. 3A. To ensure that we could compare the collectivity of the two swabs without the diluting effect getting in the way, we used a total volume of 400 µL of NW solution for both collection and releasing. The dilution volume was chosen to be 400 μL to match GenBody's COVID-19 self-test kit (Ag Home Test), which includes 400 μL of pre-filled buffer for the LFA reaction, to ensure methodological consistency in subsequent experiments. The 3DPFS used 250 µL to collect the NF and released what it had collected into a microtube containing 150 µL. The CS does not require NW solution to collect the fluid but needs it to release what it has collected for analysis, so it was dipped into a microtube containing 400 µL. Therefore, the overall volume of NW solution used for collection was equaled.

The collected samples were evaluated using both volume and color intensity measurements. To establish a reference for determining the performance of each swab in collecting NF, a standard curve (Fig. 3C) was generated by two-fold diluting the blue dye-mixed NF with a NW solution. The optical density (OD, or Absorbance at 600 nm) of the control sample, which was undiluted, was 0.34, and a standard curve was generated following the trajectory of OD = 0.3305*x* + 0.0079, where *x* represents the concentration of blue dye. Figure 3B shows the diluted dye-mixed NF ranging from undiluted to one-sixteenth diluted solution.

As depicted in Fig. 3D, both the 3DPFS and CS were able to collect all the loaded samples, but the 3DPFS retained significantly less colored sample within the swab compared to the CS, regardless of the viscosity. The color of the solution within the tube was also significantly darker for the 3DPFS than for the CS. The collected and released amounts, as well as the optical density of the collected samples for each swab, were compared and are presented in graphs (Fig. 3E–G).

Figure 3E indicates that the collection efficiency did not differ between the 3DPFS and CS, but the 3DPFS was able to release significantly more of the collected sample to the tube than the CS (Fig. 3F), resulting in approximately 4 times greater color intensity (Fig. 3G). The 3DPFS demonstrated a relatively superior performance compared to the CS, collecting 78.7% of the sample while the CS only collected 21.5%, as shown in Fig. 3G. Regardless of viscosity, the 3DPFS exhibited statistically significant performance over the CS.

The observations depicted in Fig. 3F highlight an important aspect: the loss of volume from the CS upon releasing the collected sample into the tube. This phenomenon can be attributed to the cotton surface of the swab carrying the collected nasal sample when withdrawn from the collection tube [17]. In stark contrast, the 3DPFS did not exhibit this issue, suggesting its proficiency in releasing the collected sample. This distinction is particularly noteworthy due to the differing mechanisms employed by the 3DPFS and CS. Remarkably, the 3DPFS demonstrated minimal loss, signifying its exceptional sample retention capabilities compared to conventional swabs. This unique trait translates to an augmented overall sample collectivity.

During the experimentation with NF samples, it was observed that the CS lost volume instead of adding volume to the collecting tube. This is due to the high viscosity of the NF, which coats the pores of the cotton tip and makes it difficult for the inner part of the CS to absorb the sample. When the cotton swab is soaked in a NW solution, the outer layer of the cotton tip gets wet, but the inner part of the tip remains dry. As a result, when the dry inner part of the cotton tip comes into contact with the high viscosity NF, the fluid tends to coat the pores of the cotton tip and makes it difficult for the inner part of the swab to absorb the sample. This can cause the tubes in which the CS were dipped to lose volume in the final collection, as the absorbed solution diffused with the NF sample is released along with the swab. In contrast, the 3DPFS does not absorb or soak up the sample, allowing it to easily release most of the collected fluid. This feature makes the 3DPFS a more effective tool for collecting and releasing viscous NF.

### Detection for SARS-CoV-2 in NF collected from slide glasses by 3DPFS and CS

**Fig. 4:**
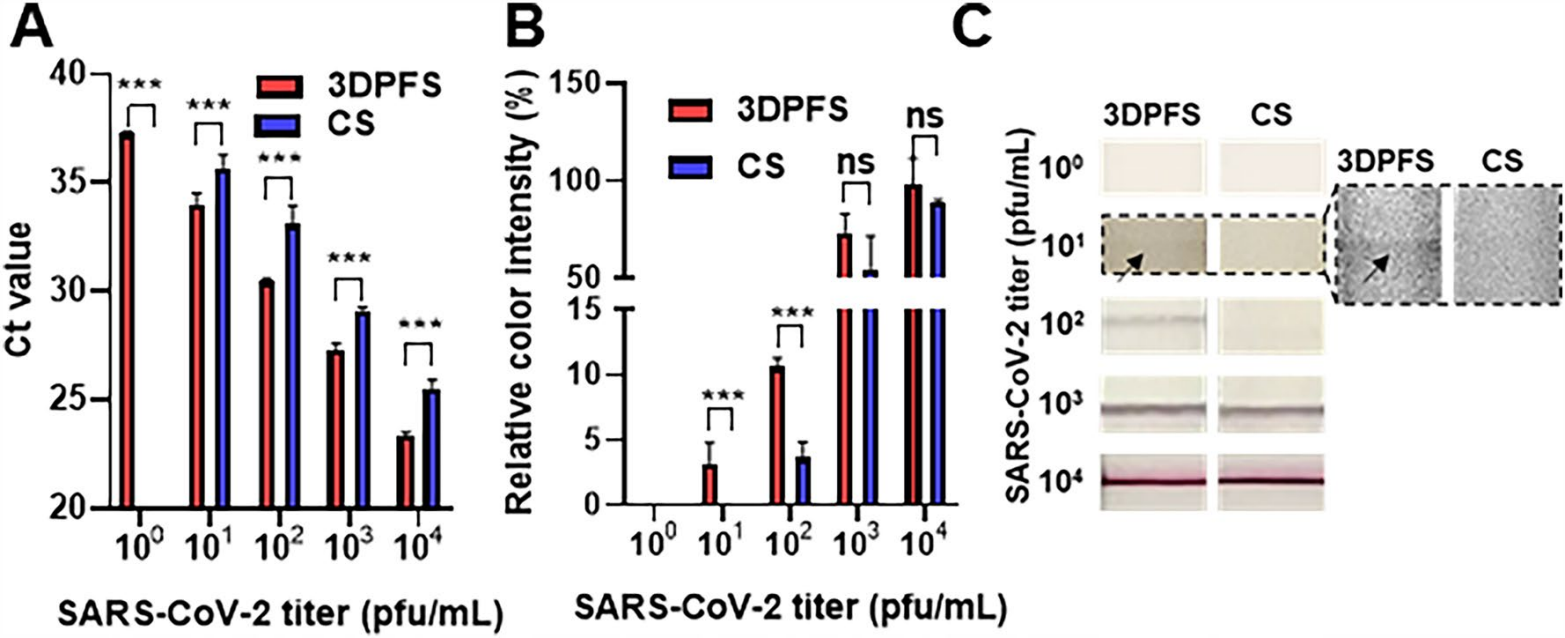
RT-qPCR and LFA for the detection of SARS-CoV-2 in NF collected by the 3DPFS and CS. **A** Schematic representation of the experiment demonstrating the collection of 100 μL of NF mixed with varying concentrations of viral lysate on a slide glass, using either 3DPFS or CS. **B** The results of RT-qPCR analysis for the collected samples. **C** The test lines of self-diagnostic kits using the collected samples. Student’s t-test was used to compare the data obtained under different conditions. Data with a *p*-value less than 0.05 were considered significant (*p < 0.05, **p < 0.01, ***p < 0.001). n = 3

To further confirm the efficacy of 3DPFS, NF containing SARS-CoV-2 lysates were tested. The virus-spiked NF on slide glasses were collected by 3DPFS and CS, respectively, and then analyzed by RT-qPCR and LFA. RT-qPCR results showed that all of the samples collected by the 3DPFS had lower C_t_ values compared to those collected by the CS (Fig. 4A, Additional file 1: Table S3). The spectrum of Ct values for negative samples can vary depending on the specific primers employed. Generally, values surpassing 35 are indicative of a negative sample [23]. For this particular study, primers of exceptional sensitivity were employed, enabling the detection of signals up to a Ct value of 37.3. Notably, the non-template control consistently exhibited a Ct value of 38 or beyond. This heightened sensitivity proved invaluable in discerning variations in swab efficacy, particularly in scenarios involving scant viral load concentrations. Especially at 10^0^ pfu/mL, the samples collected by the CS showed undetermined C_t_ value, while the samples collected by the 3DPFS showed an average C_t_ value of 37.2 ± 0.09 (*n* = 3). These results suggest that the 3DPFS is more effective than the CS in collecting and preserving virus for amplification. To quantitatively compare the performance of the 3DPFS and CS, we calculated the difference in viral RNA amplification between each sample using the ΔΔC_t_ method [24]. The 2^−ΔΔCt^ values of 4.53, 3.41, 6.11, and 3.32 were obtained for 10, 10^2^, 10^3^, and 10^4^ pfu/mL SARS-CoV-2-spiked NF samples, respectively. This indicates that 3DPFS allows us to detect SARS-CoV-2 at least 3 times more sensitive than CS.

Similarly, when LFA was performed using the virus-spiked NF samples collected by both devices (Fig. 4B, C), the 3DPFS showed higher color intensity than the cotton swab at lower concentrations. At a concentration of 10 pfu/mL, a very thin red line was observed at the test line with the sample collected by the 3DPFS, while no line was observed at the test line with the sample collected by the CS. Both RT-qPCR and LFA results showed that the 3DPFS has higher sensitivity than the CS. These results suggest that the 3DPFS is a suitable sampling method for detecting SARS-CoV-2, particularly at low concentrations in NF when compared to the CS.

### Detection for SARS-CoV-2 in NF and PNS collected from 3D printed human nose by 3DPFS and CS

**Fig. 5:**
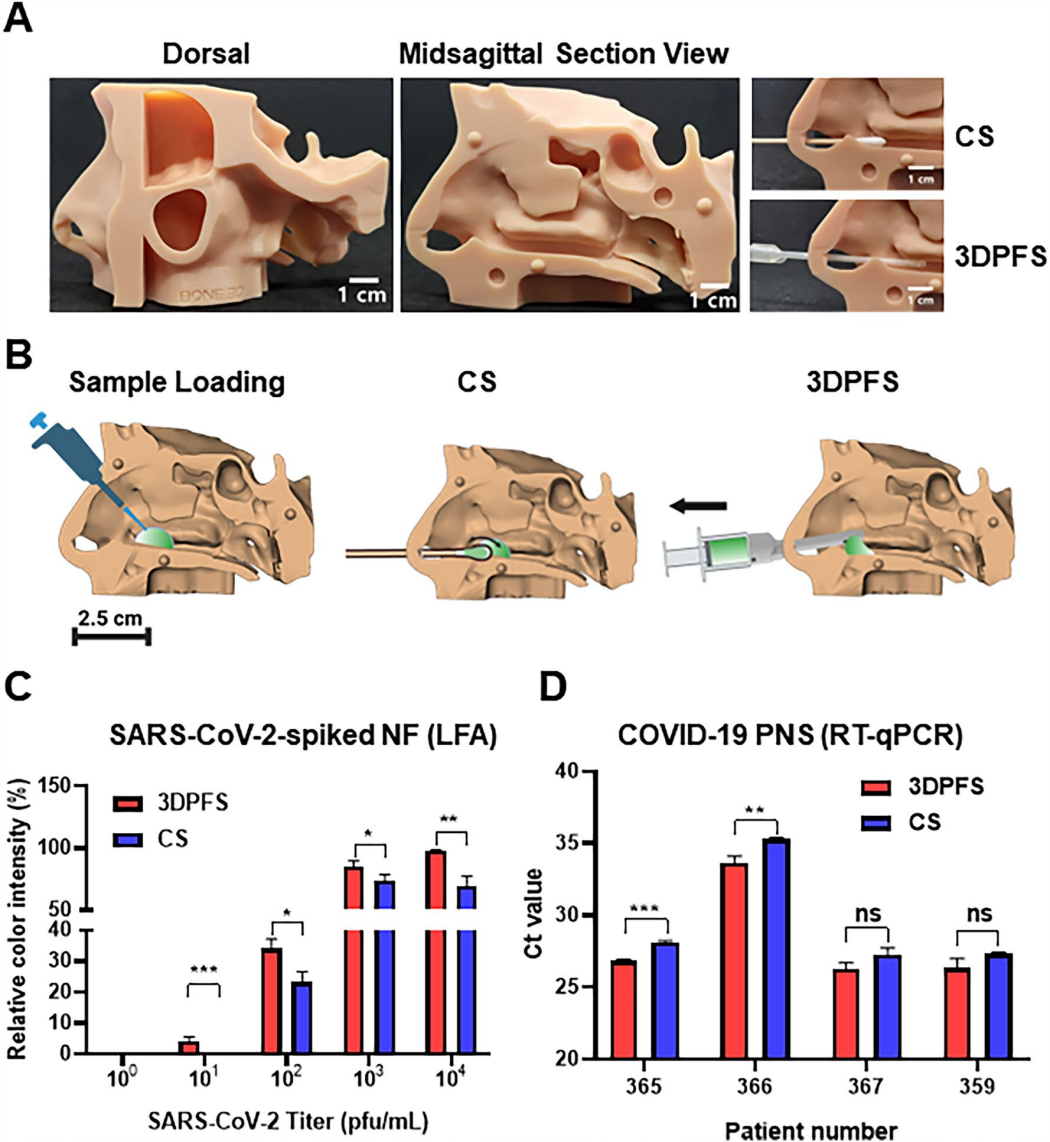
LFA and RT-qPCR detection for SARS-CoV-2 in NF and PNS collected from the 3D printed human nose by the 3DPFS and CS. **A** The 3D printed human nose and collection of NF or PNS from the nose by NF and PNS. **B** Schematic describing sample loading and collection of the sample in the nose by a pipette and either CS or 3DPFS, respectively. 100 µL of SARS-CoV-2 lysates-spiked NF at different concentrations (10^1^–10^4^ pfu/mL) or COVID-19 PNS was loaded into the nose by a pipette. **C** The results of LFA for the collected SARS-CoV-2-spiked NF from the nose 3DPFS and CS. Quantitative analysis of the test l’ne’s color intensity relative to that of the control line, using ImageJ. Student’s t-test was used to compare the data obtained under different conditions. **D** The results of RT-qPCR analysis for the collected PNS samples from the nose by 3DPFS and CS. Data with a *p*-value less than 0.05 were considered significant (*p < 0.05, **p < 0.01, ***p < 0.001). n = 3

To demonstrate the feasibility of using the 3DPFS in humans, we 3D printed a replica of the human nasal cavity as previously reported (Fig. 5A) [18]. We then loaded equal amounts of virus-spiked NF into the printed nose and collected the NF using both the 3DPFS and CS, as depicted in Fig. 5B. When the collected NF was detected by LFA, the limit of detection (LOD) in the NF collected by the 3DPFS and CS was 10 and 10^2^ pfu/mL, respectively (Fig. 5C). The results showed that the 3DPFS is more effective in collecting NF from the nose than CS.

To confirm whether 3DPFS could be used to detect actual clinical specimens, we obtained COVID-19 PNS from Gyeongsang National University College of Medicine and used them in experiments. PNS was flowed into a replica of human nose and collected using 3DPFS and CS, respectively. Viral RNA was then extracted and RT-qPCR was carried out. (Fig. 5D, Additional file 1: Table S4). The RT-qPCR results showed that all of PNS collected by the 3DPFS had lower C_t_ values than those collected by CS, implying that the amount of SARS-CoV-2 collected by 3DPFS is larger than that of CS. Note that the difference between the C_t_ values of 3DPFS and CS was the largest in the case of PNS (#366), which has a relatively high C_t_ value among the four samples. Although it is not statistically significant due to the small number of samples tested, it is assumed that 3DPFS works more effectively in samples with lower virus concentrations, where sensitivity is more critical. We also calculated viral RNA amplification using the ΔΔC_t_ method as in the previous NF result analysis. The results confirmed that the amplification difference ranged from approximately 1.94–3.18, depending on the sample. By using the 3DPFS developed in this study, SARS-CoV-2 in PNS can be detected at least twice more sensitive than CS. Meanwhile, four COVID-19 negatively diagnosed PNS samples provided undetermined C_t_ values after collecting by both 3DPFS and CS (data not shown).

Overall, these findings showed that the 3DPFS has several benefits compared to CS in collecting and releasing NF, which leads to increased sensitivity and accuracy in detecting SARS-CoV-2 through LFA and RT-qPCR.

### User survey for easiness and comfortability of 3DPFS and CS

Additionally, a survey of 28 volunteers found that approximately 90% of users preferred the 3DPFS over the CS because the 3DPFS caused less pain and discomfort, indicating that it may be a promising alternative for nasal sampling. The survey results are presented in Additional file 1: Fig. S6 and Table S5. This suggests that the 3DPFS represents a significant improvement over CS for COVID-19 testing, enabling the collection of more NF from the nose with relatively less discomfort. Furthermore, the innovative approach may have implications for the development of more effective and user-friendly testing methods for SARS-CoV-2 and other respiratory viruses.

## Conclusions

In summary, the use of a 3DPFS has demonstrated superior capabilities for collecting and releasing NF compared to the traditional cotton swabs, resulting in an improved sensitivity for detecting SARS-CoV-2 by both LFA and RT-qPCR. The increased sensitivity of the 3DPFS suggests its potential use in self-diagnostic tools to detect the virus at early and late stages of infection, enabling prompt treatment and reducing the spread of the virus. The 3DPFS is also cost-effective, easily mass-produced using DLP printing, and can be attached to low volume commercial syringes. The design features of the 3DPFS, including protrusions for mixing nasal samples with NW solution and a large inlet to reduce required sampling pressure, make it a versatile tool for collecting samples beyond viruses. The 3DPFS's simple motion for fluid collection within the nose may lead to its development into an automated system for non-face-to-face collecting machines during pandemics. While further studies are necessary to evaluate the 3DPFS's capabilities for other specimens, the results suggest its potential for improved sample collection in general.

### Supplementary Information

**Additional file 1: Table S1.** Vat photopolymerization parameters for 3DPFS fabrication. **Table S2.** Vat photopolymerization parameters for 3D printed human nose. **Table S3.** Detection of inactivated SARS-CoV-2-spiked NF on a slide glass. **Table S4.** Detection of SARS-CoV-2 from PNS loaded on a 3D printed human nose model. **Table S5.** Survey results for the comparison of CS and 3DPFS, illustrated in table. The colors indicate the overall preference, with red indicating a preference for 3DPFS and blue indicating a preference for CS. **Fig S1.** 3D Printed human face made of the standard beige photopolymer resin. The equal amounts of SARS-CoV-2-spiked NF or COVID-19 PNS were loaded into the MT region of the printed nose. **Fig S2.** In the context of LFA using ImageJ, testing regions were carefully chosen to quantify the color intensity of each line. To minimize the influence of any extraneous background signals, a trendline was drawn and subsequently utilized to isolate the region of interest corresponding to the peak color intensity. This region was then measured and recorded as the color intensity of the line. The color intensities of the 3DPFS and CS were represented by blue and orange lines, respectively. **Fig S3.** Questionnaire used in the survey to assess participants' pain, discomfort, and preference between CS and the 3DPFS for COVID-19 testing. The questionnaire included the following questions: (1) Please rate the relative level of pain experienced during the test; (2) Please rate the level of after effects or discomfort you experienced after the test; (3) Which swab do you prefer for COVID-19 testing, the CS or the 3DPFS. **Fig S4.** The generated mesh and the CFD simulation results showing volume fraction, fluid velocity, and sampling pressure for 1L, 1S, and 2S cases. **Fig S5.** The generated mesh and the CFD simulation results showing volume fraction, fluid velocity and sampling pressure for 6S case. No sample or liquid solution were introduced from the inlet other than air. **Fig S6.** Survey results for the comparison of 3DPFS and CS, illustrated in terms of participants' reported levels of pain and discomfort, as well as their preferences between the two swabs. (A, B) The x-axis represents the magnitude of pain or discomfort, with positive values indicating greater discomfort associated with the CS and negative values representing more pain or discomfort attributed to the 3DPFS. (C) Participants' preference for either swab.

## Data Availability

Vat photopolymerization parameters for 3DPFS fabrication, Vat photopolymerization parameters for 3D printed human nose, Detection of inactivated SARS-CoV-2-spiked NF on a slide glass, Detection of SARS-CoV-2 from PNS loaded on a 3D printed human nose model, Survey results for the comparison of CS and 3DPFS, illustrated in table, 3D Printed human face made of the standard beige photopolymer resin, LFA using ImageJ, Questionnaire used in the survey to assess participants' pain, discomfort, and preference between CS and the 3DPFS for COVID-19 testing, The generated mesh and the CFD simulation results showing volume fraction, fluid velocity, and sampling pressure for 1L, 1S, and 2S cases, The generated mesh and the CFD simulation results showing volume fraction, fluid velocity and sampling pressure for 6S case, Survey results for the comparison of 3DPFS and CS, illustrated in terms of participants’ reported levels of pain and discomfort, as well as their preferences between the two swabs (PDF). This material is available free of charge via the Internet at http://nanoconvergencejournal.springeropen.com.

## Abbreviations

3DPFS: 3D printed fluidic swab
CS: Cotton swab
NW: Nasal wash
NP: Nasopharyngeal
MT: Mid-turbinate
NF: Nasal fluid
DLP: Digital light processing
pfu: Plaque forming units
RNA: Ribonucleic acid
COVID-19: Coronavirus disease 2019
RT-qPCR: Reverse transcription-quantitative polymerase chain reaction
LFA: Lateral flow assay
PNS: Patient nasal samples
C_t_: Threshold cycle
3D: Three-dimensional
TCEP: Tris(2-carboxyethyl)phosphine hydrochloride
EDTA: Ethylenediaminetetraacetic acid
UV: Ultraviolet
CFD: Computational fluid dynamics
ORF: Open reading frame
