# Supplementary material for: 3D printed fluidic swab for COVID-19 testing with improved diagnostic yield and user comfort
Source: Nano Converg. 2023 Sep 16;10:45. doi: 10.1186/s40580-023-00393-3 (PMC10505115; doi:10.1186/s40580-023-00393-3)
Supplement: Supplementary file 1 — Additional file 1: Table S1. Vat photopolymerization parameters for 3DPFS fabrication. Table S2. Vat photopolymerization parameters for 3D printed human nose. Table S3. Detection of inactivated SARS-CoV-2-spiked NF on a slide glass. Table S4. Detection of SARS-CoV-2 from PNS loaded on a 3D printed human nose model. Table S5. Survey results for the comparison of CS and 3DPFS, illustrated in table. The colors indicate the overall preference, with red indicating a preference for 3DPFS and blue indicating a preference for CS. Fig S1. 3D Printed human face made of the standard beige photopolymer resin. The equal amounts of SARS-CoV-2-spiked NF or COVID-19 PNS were loaded into the MT region of the printed nose. Fig S2. In the context of LFA using ImageJ, testing regions were carefully chosen to quantify the color intensity of each line. To minimize the influence of any extraneous background signals, a trendline was drawn and subsequently utilized to isolate the region of interest corresponding to the peak color intensity. This region was then measured and recorded as the color intensity of the line. The color intensities of the 3DPFS and CS were represented by blue and orange lines, respectively. Fig S3. Questionnaire used in the survey to assess participants' pain, discomfort, and preference between CS and the 3DPFS for COVID-19 testing. The questionnaire included the following questions: (1) Please rate the relative level of pain experienced during the test; (2) Please rate the level of after effects or discomfort you experienced after the test; (3) Which swab do you prefer for COVID-19 testing, the CS or the 3DPFS. Fig S4. The generated mesh and the CFD simulation results showing volume fraction, fluid velocity, and sampling pressure for 1L, 1S, and 2S cases. Fig S5. The generated mesh and the CFD simulation results showing volume fraction, fluid velocity and sampling pressure for 6S case. No sample or liquid solution were introduced from the inlet other than air. Fig [file 40580_2023_393_MOESM1_ESM.docx]

Supporting Information

**3D Printed Fluidic Swab for COVID-19 Testing with Improved Diagnostic Yield and User Comfort**

Joochan Kim^1^, Jaehyung Jeon^1^, Hyowon Jang^2^, Youngkwang Moon^1^, Abdurhaman Teyib Abafogi^1^, Danny van Noort^3^, Jinkee Lee^1,4^, Taejoon Kang^2,5*^, Sungsu Park^1,4*^

^1^ School of Mechanical Engineering, Sungkyunkwan University (SKKU), Suwon, Korea

^2^ Bionanotechnology Research Center, Korea Research Institute of Bioscience and Biotechnology (KRIBB), Daejeon, Korea

^3^ Division of Biophysics and Bioengineering, IFM, Linköping University, Linköping, Sweden

^4^ Department of Biophysics, Institute of Quantum Biophysics (IQB), Sungkyunkwan University (SKKU), Suwon, Korea

^5^ School of Pharmacy, Sungkyunkwan University (SKKU), Suwon, Korea

* Corresponding author: Taejoon Kang; Sungsu Park

Tel.: +82-42-879-8453; +82-31-290-7431

E-mail address: kangtae-joon@kribb.re.kr (T. Kang); nanopark@skku.edu (S. Park)

**Table S1.** Vat photopolymerization parameters for 3DPFS fabrication.

| **Parameters (CUKH010C, IM1)** | |
| --- | --- |
| Power (W/m^2^) | 350 |
| Printing plate size (mm × mm) | 96 × 54 |
| Layer thickness (mm) | 0.1 |
| Motor speed (mm/s) | 1 |
| Motor speed adjustable height (mm) | 3 |
| Initial layer waiting time (s) | 6 |
| Layer waiting time (s) | 4 |
| Initial exposure time (s) | 5 |
| Exposure time (s) | 1.4 |

**Table S2.** Vat photopolymerization parameters for 3D printed human nose.

| **Parameters (3DK83B, TM200)** | |
| --- | --- |
| Power (W/m^2^) | 350 |
| Printing plate size (mm × mm) | 96 × 54 |
| Layer thickness (mm) | 0.1 |
| Motor speed (mm/s) | 1 |
| Motor speed adjustable height (mm) | 3 |
| Initial layer waiting time (s) | 5 |
| Layer waiting time (s) | 3 |
| Initial exposure time (s) | 15 |
| Exposure time (s) | 3.5 |

**Table S3.** Detection of inactivated SARS-CoV-2-spiked NF on a slide glass.

| **3DPFS** | | | | | |
| --- | --- | --- | --- | --- | --- |
| Titer (pfu/mL) | 10^4^ | 10^3^ | 10^2^ | 10^1^ | 10^0^ |
| Cycle Threshold (C_t_) | 23.36 ± 0.15 | 27.31 ± 0.30 | 30.49 ± 0.12 | 33.97 ± 0.54 | 37.27 ± 0.09 |
| True Positive Rate (%) | 100 | 100 | 100 | 100 | 33 |
| False Negative Rate (%) | 0 | 0 | 0 | 0 | 66 |
| **CS** | | | | | |
| Titer (pfu/mL) | 10^4^ | 10^3^ | 10^2^ | 10^1^ | 10^0^ |
| Cycle Threshold (C_t_) | 25.54 ± 0.41 | 29.08 ± 0.18 | 33.10 ± 0.85 | 35.70 ± 0.57 | Und |
| True Positive Rate (%) | 100 | 100 | 100 | 83 | 0 |
| False Negative Rate (%) | 0 | 0 | 0 | 17 | 100 |

**Table S4.** Detection of SARS-CoV-2 from PNS loaded on a 3D printed human nose model.

| **3DPFS** | | | | |
| --- | --- | --- | --- | --- |
| Patient # | 359 | 365 | 366 | 377 |
| Cycle Threshold (C_t_) | 26.27 ± 0.45 | 26.82 ± 0.11 | 33.63 ± 0.50 | 26.35 ± 0.65 |
| True Positive Rate (%) | 100 | 100 | 100 | 100 |
| False Negative Rate (%) | 0 | 0 | 0 | 0 |
| **CS** | | | | |
| Patient # | 359 | 365 | 366 | 377 |
| Cycle Threshold (C_t_) | 27.24 ± 0.51 | 28.06 ± 0.16 | 35.30 ± 0.09 | 27.31 ± 0.10 |
| True Positive Rate (%) | 100 | 100 | 100 | 100 |
| False Negative Rate (%) | 0 | 0 | 0 | 0 |

**Table S5.** Survey results for the comparison of CS and 3DPFS, illustrated in table. The colors indicate the overall preference, with red indicating a preference for 3DPFS and blue indicating a preference for CS.


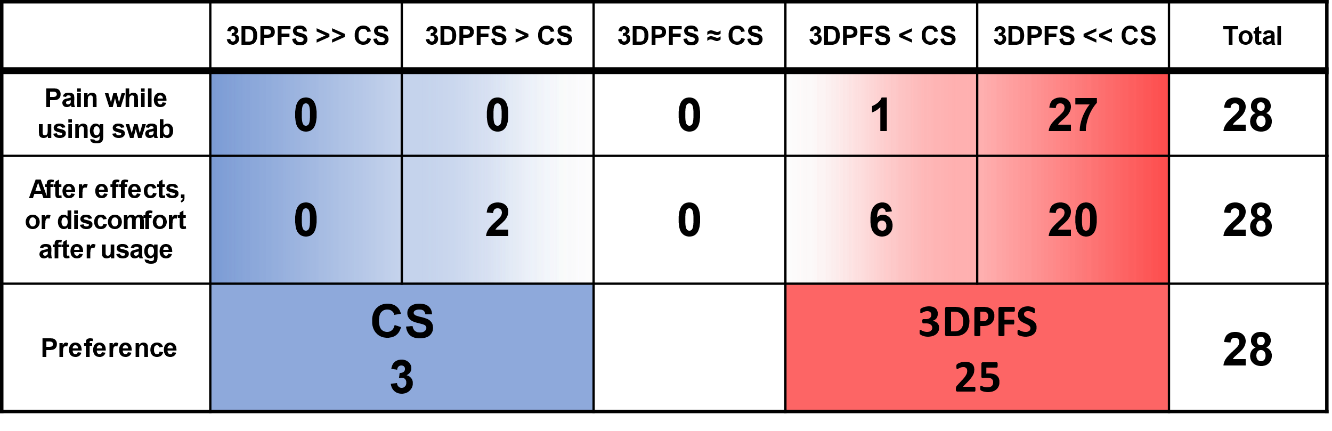


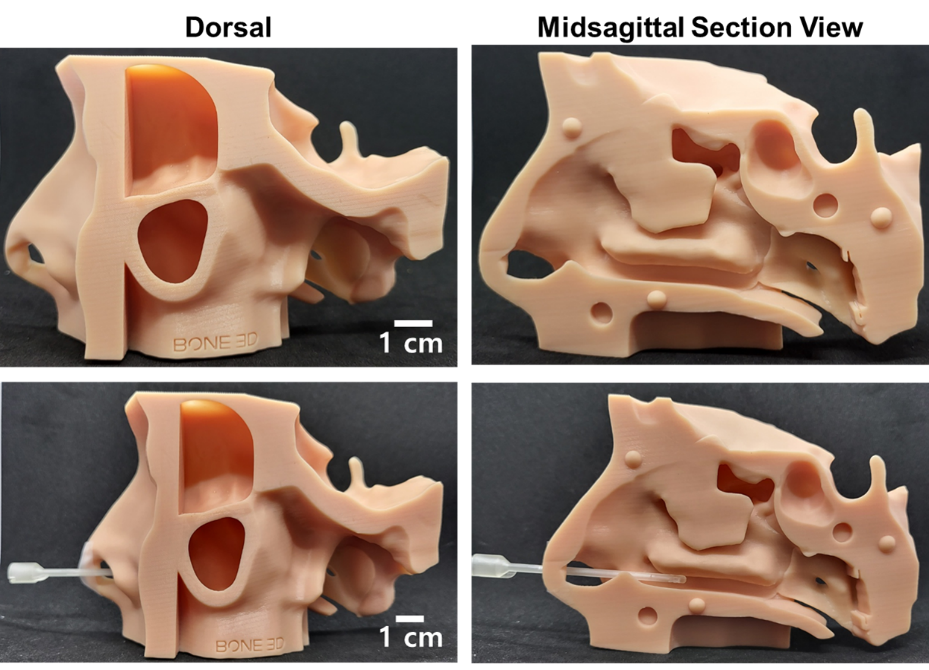


**Fig. S1.** 3D Printed human face made of the standard beige photopolymer resin. The equal amounts of SARS-CoV-2-spiked NF or COVID-19 PNS were loaded into the MT region of the printed nose.


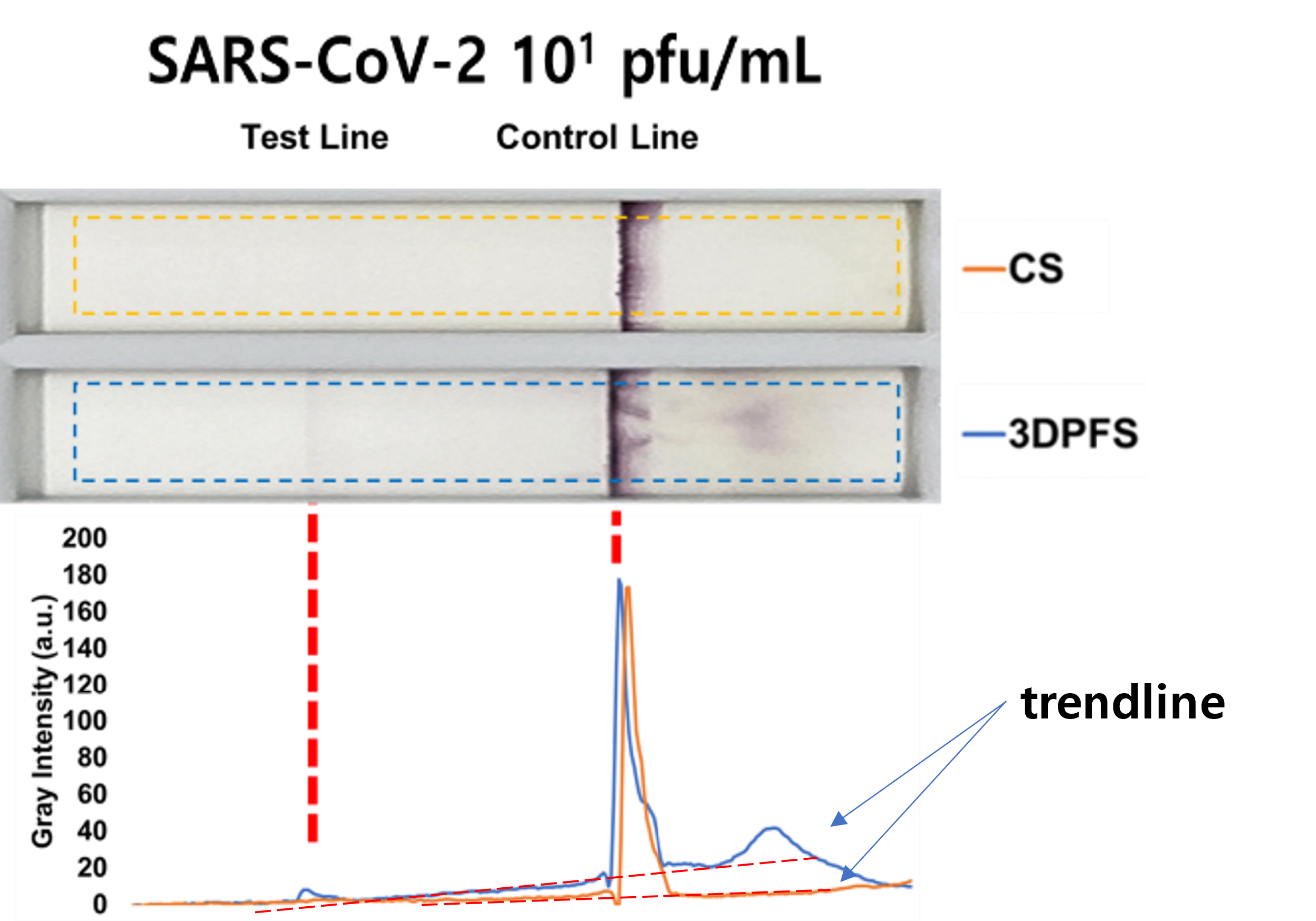


**Fig. S2.** In the context of LFA using ImageJ, testing regions were carefully chosen to quantify the color intensity of each line. To minimize the influence of any extraneous background signals, a trendline was drawn and subsequently utilized to isolate the region of interest corresponding to the peak color intensity. This region was then measured and recorded as the color intensity of the line. The color intensities of the 3DPFS and CS were represented by blue and orange lines, respectively.


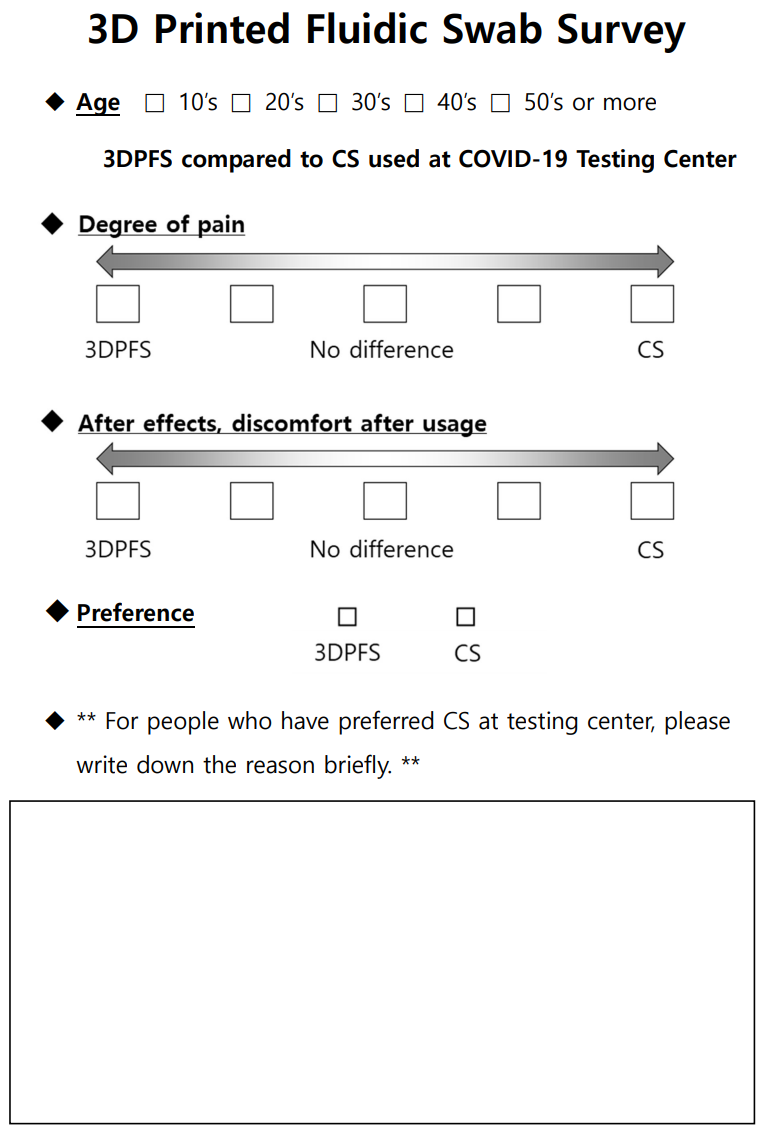


**Fig. S3.** Questionnaire used in the survey to assess participants' pain, discomfort, and preference between CS and the 3DPFS for COVID-19 testing. The questionnaire included the following questions: (1) Please rate the relative level of pain experienced during the test; (2) Please rate the level of after effects or discomfort you experienced after the test; (3) Which swab do you prefer for COVID-19 testing, the CS or the 3DPFS.


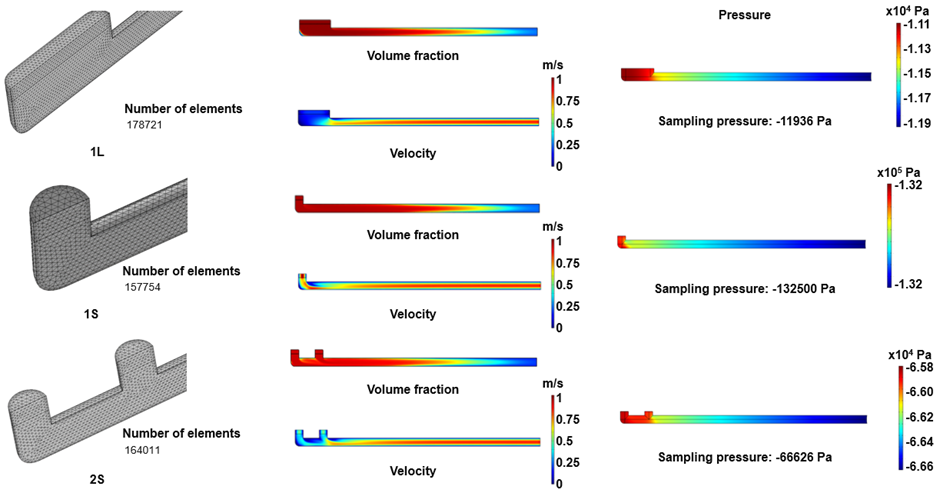


**Fig. S4.** The generated mesh and the CFD simulation results showing volume fraction, fluid velocity, and sampling pressure for 1L, 1S, and 2S cases.


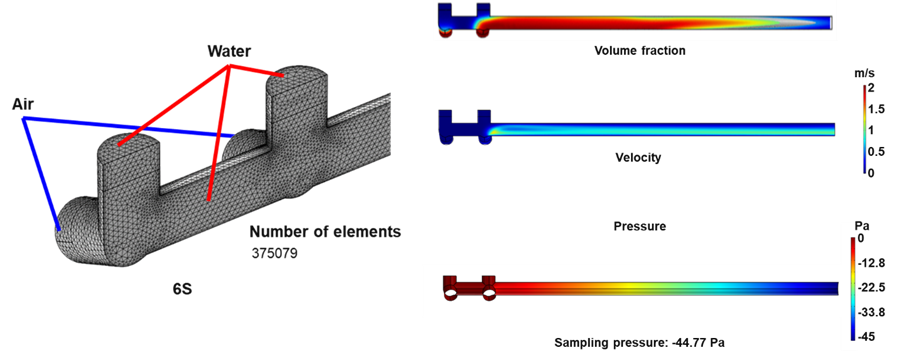


**Fig. S5.** The generated mesh and the CFD simulation results showing volume fraction, fluid velocity and sampling pressure for 6S case. No sample or liquid solution were introduced from the inlet other than air.


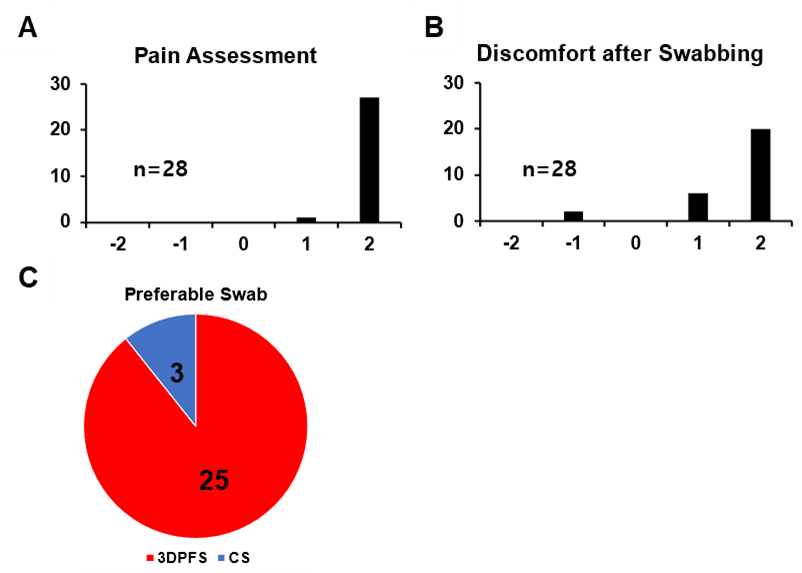


**Fig. S6.** Survey results for the comparison of 3DPFS and CS, illustrated in terms of participants' reported levels of pain and discomfort, as well as their preferences between the two swabs. (A, B) The x-axis represents the magnitude of pain or discomfort, with positive values indicating greater discomfort associated with the CS and negative values representing more pain or discomfort attributed to the 3DPFS. (C) Participants' preference for either swab.
